# Assessing Mental Illness Risk Among North Korean Refugees and Immigrants Resettled in South Korea

**DOI:** 10.1001/jamanetworkopen.2022.36751

**Published:** 2022-10-19

**Authors:** Rugyeom Lee, Sang Min Lee, Minha Hong, In-Hwan Oh

**Affiliations:** 1 Department of Preventive Medicine, Kyung Hee University School of Medicine, Seoul, South Korea; 2 Department of Psychiatry, Kyung Hee University School of Medicine, Seoul, South Korea; 3 Department of Psychiatry, Myongji Hospital, Hanyang University College of Medicine, Goyang, South Korea

## Abstract

**Importance:**

The global increase in the numbers of refugees and immigrants has made mental health a major public concern. Although they are divided, North and South Korea share a common ethnic heritage.

**Objective:**

To investigate mental illness risk among North Korean immigrants living in South Korea and compare it with that of the general South Korean population.

**Design, Setting, and Participants:**

This retrospective, population-based cohort study was based on National Health Insurance claims data from 2007 to 2019, and North Korean immigrants and refugees (NKIRs) and age-matched and sex-matched members of the general population (GP) were included with a 1:3 ratio. Data were analyzed from March 2022 to August 2022.

**Exposure:**

Emigration from North Korea to South Korea.

**Main Outcomes and Measures:**

Mental illness incidence and risk through a Cox proportional risk model adjusted for sex, age, disability, region, Charlson Comorbidity Index score, and insurance premium percentile.

**Results:**

A total of 37 209 NKIRs and 111 627 members of the GP were enrolled. The final analysis examined 112 851 individuals (73 238 [64.9%] female, median [IQR] age 34 [19-47] years), with 90 235 (80.0%) in the GP and 22 616 (20.0%) in the NKIR group. The propensity score–matching population numbered 24 532 in total, with 12 266 each in the NKIR and GP groups. NKIR patients had a higher mental illness risk than the GP (hazard ratio [HR], 2.12; 95% CI, 2.04-2.21). The multivariable adjusted HRs (95% CI) for developing mental disorders were 4.91 (3.59-6.71) for posttraumatic stress disorder, 3.10 (2.90-3.30) for major depressive disorder, 2.27 (2.11-2.44) for anxiety and panic disorder, 2.03 (1.58-2.60) for bipolar affective disorder, 1.85 (1.53-2.24) for alcohol use disorder, and 1.89 (1.46-2.45) for schizophrenia.

**Conclusions and Relevance:**

In this study, NKIR individuals showed a significantly higher risk of developing most mental disorders. Interventional strategies can prevent psychiatric morbidity and ensure adequate care for this population.

## Key Points

**Question:**

What is the mental illness risk among North Korean refugees and immigrants settled in South Korea compared with that of South Korean citizens?

**Findings:**

In this cohort study of a propensity score-matched population of 24 532 individuals, North Korean refugees and immigrants showed a significantly higher risk of developing major depressive disorder, anxiety and panic disorder, alcohol use disorder, and posttraumatic stress disorder compared with South Korean citizens.

**Meaning:**

These findings suggest the increased risk of psychiatric disorders was significant in disorders that were attributed to environmental stressors, such as major depressive disorder, anxiety and panic disorder, alcohol use disorder, and posttraumatic stress disorder, rather than in psychiatric disorders with strong genetic heritability.

## Teaser

This cohort study evaluates the risk of mental illness among North Korean refugees and immigrants resettled in South Korea compared with South Korean citizens.

## Introduction

### Background

Although deriving exact relevant mental health–related figures for immigrants is difficult,^1^ the topic deserves study because the number of persons forcibly displaced across national borders worldwide was 34 million in 2019, which was twice the corresponding number in 2000,^2^ as indicated by the International Migration Report.^3^ Existing literature^4,5^ has shown that immigrants and refugees are vulnerable to mental health disorders. One of the largest meta-analyses,^6^ which included 161 articles and examined 81 833 refugees from over 40 countries, reported higher rates of posttraumatic stress disorder (PTSD) and depression in this group, even after adjusting for methodological factors. A systematic review^7^ of depression and anxiety among refugees showed that their refugee status had a major impact on the mental health of this population.

The Korean Peninsula currently has the only divided single-ethnicity country in the world, and it is currently in a state of ceasefire. Although their language and culture are shared, because the socioeconomic levels of North Korea (officially the Democratic People's Republic of Korea) and South Korea (officially the Republic of Korea) differ substantially, most people who leave North Korea settle in South Korea. According to statistics from the Ministry of Unification, the number of North Korean immigrants and refugees (NKIRs) at the end of 2020 was 33 752, and the number of North Korean immigrants reported by the United Nations was 49 549, indicating that 68% of NKIRs were absorbed into South Korea.^8^ In South Korea, the government officially provides settlement support for NKIRs, and their medical insurance is managed under a separate management code. Therefore, accurate medical claims data on NKIRs can be obtained for analysis. Nevertheless, any epidemiologic evidence for determining mental illness risk among NKIR groups has remained underexamined thus far because, to our knowledge, no studies have analyzed these issues in the entire population. Existing studies on NKIRs have targeted limited samples of dozens or hundreds of people of a specific age or sex, and most of these have not included a control group.^8^ Using national insurance data, this study sought to examine mental illness risk among NKIRs in comparison to that of the general population (GP).

## Methods

### Study Design and Data Sources

This study followed the Strengthening the Reporting of Observational Studies in Epidemiology (STROBE) reporting guideline. The Korea University institutional review board approved this study and waived the need for informed consent because it follows the Korean guidelines for deidentification of personal data. National Health Insurance Service (NHIS) claims data for the period of 2007 to 2019 were used to compare mental illness occurrence between the GP and NKIRs. The data include numerous variables such as demographics, claims, information on death, insurance premium level, and disability. Insurance premiums were divided into 21 (0-20th percentile) divisions according to monthly income levels in the NHIS, and this variable was applied as a continuous variable for analysis. It was used as an indicator of individual income level. The *International Statistical Classification of Diseases and Related Health Problems, Tenth Revision (ICD-10)* code was used for disease classification (Table 1), and the initial study population data for all NKIRs in the claims data were requested. The control group, the GP group, was determined by extracting a 1:3 ratio. Sex and age were matched and extracted according to the NHIS data provision standards. The current study conducted analysis using propensity score matching (PSM), wherein NKIR and GP were matched 1:1. The variables used for matching were sex, age, insurance premium quartile, region (metropolitan area: Seoul, Gyeonggi, and Incheon; nonmetropolitan area: other regions), and Charlson Comorbidity Index (CCI) score.

**Table 1.** *ICD-10* Codes Corresponding to the Mental Disorder

Mental disorder	*ICD-10* code
Schizophrenia	F20, F21, F22, F23, F24, F25, F26, F27, F28, F29
Alcohol use disorders	F10, X45, Q860
Substance use disorders	
Opioid use disorders	F11
Cocaine use disorders	F14
Amphetamine use disorders	F15
Cannabis use disorders	F12
Other drug disorders	F13, F16, F18, F19, X41, X42, X49
Major depressive disorders	F32, F33
Dysthymia	F341
Bipolar affective disorder	F30, F31
Anxiety and panic disorder	F40, F41
Obsessive-compulsive disorder	F42
Posttraumatic stress disorder	F431
Dissociative (conversion) disorders	F44
Eating disorders	F50
Autistic disorder	F84
Attention-deficit/hyperactivity disorder	F90
Conduct disorder	F91, F92
Idiopathic intellectual disability	F70, F71, F72, F73, F78, F79
Borderline personality disorder	F60
Other mental disorders	F04, F05, F06, F07, F09, F17, F38, F39, F43, F45, F46, F47, F48, F51, F52, F53, F54, F55, F56, F57, F58, F59, F61, F62, F63, F64, F65, F66, F68, F69, F80, F81, F82, F83, F88, F93, F94, F95, F98

Abbreviation: *ICD-10*, *International Statistical Classification of Diseases and Related Health Problems, Tenth Revision*.

### Participants

The study participants were selected according to the following criterion: they had made no mental illness–related claims for hospitalization or outpatient care for 3 years after the year in which their credentials were first registered. A 3-year washout period, including the index year, was provided for participants registered between 2007 and 2016. The observation period was determined to last from the index year until December 31, 2019, when the study period ended, the patient died, or mental illness occurred, whichever occurred first (Figure). Excluding participants who already had mental illness, died during the washout period, or had missing variables during the observation period, the final number of people who showed all the variables were included in the analysis. The mean (SD) observation periods were 2242 (1165) days (6.1 years) for the NKIR group and 3158 (950) days (8.6 years) for the GP group. Regarding PSM, the mean (SD) observation periods were 2450 (1094) and 3185 (913) days for the NKIR and GP groups, respectively (eTable 1 in the Supplement).

**Figure.**
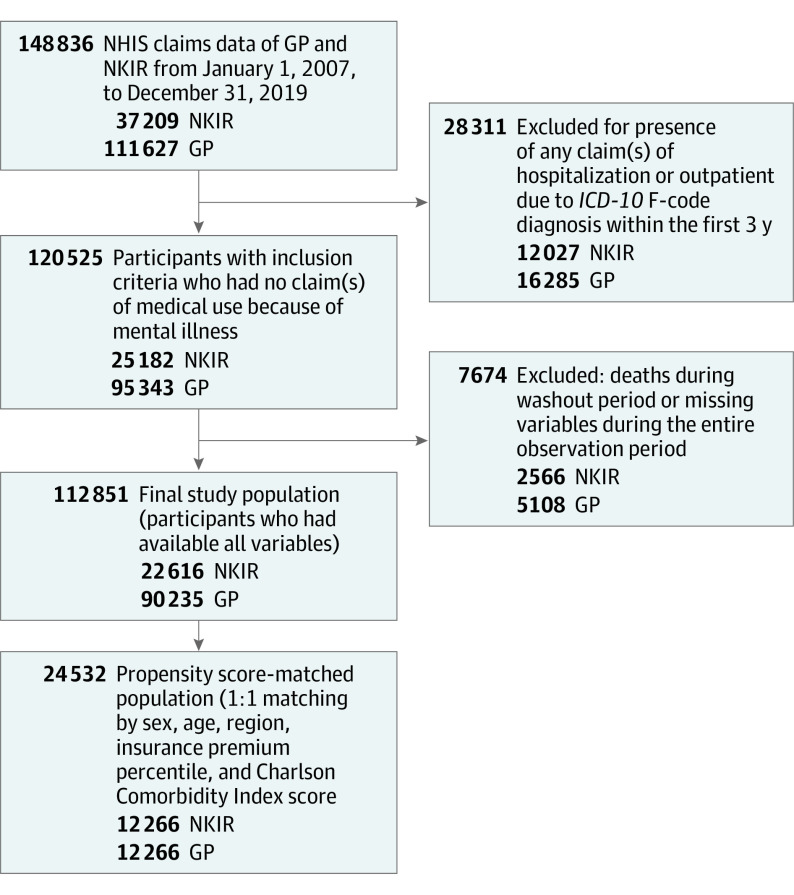
Flow Diagram of Selection Process for Participants GP indicates general population; *ICD-10*, *International Statistical Classification of Diseases and Related Health Problems, Tenth Revision*; NHIS, National Health Insurance Service; NKIR, North Korean immigrants and refugees.

### Statistical Analysis

Descriptive statistics (means and frequencies) were used for characterizing the demographic variables. The χ^2^ test was used for comparing the GP and NKIR groups. Mental illness occurrence was defined as the presence of hospitalization or outpatient status claims under any *ICD-10* F code diagnosis. The Cox proportional risk model was applied and presented in the form of hazard ratios (HRs) and 95% CIs. Individual mental illnesses’ risks were estimated. The Cox proportional hazard model was adjusted for confounding factors, including sex, age, disability, region, CCI score, and insurance premium percentile. The survival probability was estimated for each group (eFigure in the Supplement), and the differences between groups were evaluated using the log-rank test (χ^2^_1_ = 2722.9; *P* < .01). All statistical analyses were performed using the SAS Enterprise Guide tool version 7.1 provided by the NHIS (SAS Institute). Data were analyzed from March 2022 to August 2022.

## Results

Participants’ baseline characteristics are described in Table 2, before and after PSM. The initial study population comprised 37 209 NKIR and 111 627 GP. The final participants were 112 851 individuals, with 90 235 (80.0%) in the GP group and 22 616 (20.0%) in the NKIR group (73 238 [64.9%] female, median [IQR] age 34 [19-47] years). The PSM population numbered 24 532 in total, with 12 266 each in the NKIR and GP groups. The HRs for mental illness occurrence, which were adjusted for age, sex, insurance premium percentile, region, CCI score, and presence of disability, are shown in Table 3. The number of mental illness occurrences was 15 348 (from among 112 851 individuals in the original cohort). The HR for mental illness occurrence in NKIR (from among the original cohort) was 2.12 (95% CI, 2.04-2.21), and that in the PSM cohort was 1.63 (95% CI, 1.52-1.75) relative to that in the GP cohort (*P* < .001).

**Table 2.** Baseline Characteristics of Study Participants Before and After Propensity Score Matching

Characteristic	Original Participants, No. (%)		*P* value	Propensity score matching Participants, No. (%)		*P* value
	GP (n = 90 235)	NKIR (n = 22 616)		GP (n = 12 266)	NKIR (n = 12 266)	
Age group, y						
0-9	9066 (10.0)	3107 (13.7)	<.001	1126 (9.2)	1126 (9.2)	>.99
10-19	12 744 (14.1)	3904 (17.3)		2058 (16.8)	2058 (16.8)	
20-29	12 672 (14.0)	3299 (14.6)		1860 (15.1)	1860 (15.1)	
30-39	17 874 (1938)	4610 (20.4)		2831 (23.1)	2831 (23.1)	
40-49	20 280 (22.5)	4401 (19.5)		2811 (22.9)	2811 (22.9)	
50-59	12 496 (13.8)	2204 (9.7)		1345 (11.0)	1345 (11.0)	
60-69	2938 (3.3)	538 (2.4)		159 (1.3)	159 (1.3)	
70-79	1658 (1.8)	386 (1.7)		61 (0.5)	61 (0.5)	
≥80	507 (0.6)	167 (0.7)		15 (0.1)	15 (0.1)	
Age continuous, mean (SD), y	34.4 (17.3)	31.3 (17.5)	<.001	32.1 (15.3)	32.1 (15.3)	>.99
Sex						
Male	30 712 (34.0)	8901 (39.4)	<.001	4699 (38.3)	4699 (38.3)	>.99
Female	59 523 (66.0)	13 715 (60.6)		7567 (62.7)	7567 (62.7)	
Insurance premium level						
Low (0-6th)	22 480 (24.9)	15 066 (66.6)	<.001	5782 (47.1)	5782 (47.1)	>.99
Middle (7-13th)	33 746 (37.4)	6054 (26.8)		4669 (38.1)	4669 (38.1)	
High (14-20th)	34 009 (37.7)	1496 (6.6)		1815 (14.8)	1815 (14.8)	
Disability						
Yes	1818 (2.0)	738 (3.3)	<.001	329 (2.7)	243 (2.0)	<.001
No	88 417 (98.0)	21 878 (96.7)		11937 (97.3)	12023 (98.0)	
Region						
Metropolitan area	46 992 (52.1)	14 694 (65.0)	<.001	7483 (61.0)	7483 (61.0)	>.99
Nonmetropolitan area	43 243 (47.9)	7922 (35.0)		4783 (39.0)	4783 (39.0)	
Charlson Comorbidity Index score continuous, mean (SD)	1.55 (1.7)	1.78 (1.9)	<.001	1.4 (1.4)	1.4 (1.4)	>.99

Abbreviations: GP, general population; NKIR, North Korean immigrants and refugees.

**Table 3.** Factors Associated With Occurrence of Mental Illness Before and After Propensity Score Matching

Characteristic	Original				Propensity score matching			
	Incidence of mental disorder, No. of cases	Participants, No.	HR (95% CI)	*P* value	Incidence of mental disorder, No. of cases	Participants, No.	HR (95% CI)	*P* value
Group								
General population	10 737	90 235	1.00 [Reference]	NA	1465	12 266	1.00 [Reference]	NA
North Korean immigrants and refugees	4651	22 616	2.12 (2.04-2.21)	<.001	1766	12 266	1.63 (1.52-1.75)	<.001
Age, per 1 y	15 388	112 851	0.99 (0.99-0.99)	<.001	3231	24 532	0.99 (0.99-0.99)	.008
Sex								
Male	4337	39 613	1.00 [Reference]	NA	951	9398	1.00 [Reference]	NA
Female	11 051	73 238	1.41 (1.36-1.46)	<.001	2290	15 134	1.43 (1.32-1.55)	<.001
Insurance premium level, per 1 percentile	15 388	112 851	0.98 (0.98-0.98)	<.001	3231	24 532	0.98 (0.98-0.99)	<.001
Disability								
No	14 688	110 295	1.00 [Reference]	NA	3077	23 960	1.00 [Reference]	NA
Yes	700	2556	1.79 (1.65-1.94)	<.001	164	572	2.06 (1.75-2.43)	<.001
Region								
Metropolitan area	8609	61 686	1.00 [Reference]	NA	1961	14 966	1.00 [Reference]	NA
Nonmetropolitan area	6779	51 165	0.99 (0.96-1.02)	.57	1280	9566	1.01 (0.94-1.09)	.74
Charlson Comorbidity Index score, per 1 score	15 388	112 851	1.08 (1.07-1.09)	<.001	3231	24 532	1.13 (1.11-1.16)	<.001

Abbreviation: NA, not applicable.

### Differences in Mental Illness Occurrence Between NKIR and GP

The mental illness risks of GP and NKIR were analyzed (Table 4). The HRs for the individual mental illnesses included in the analyses are shown in eTable 2 in the Supplement. The HRs and 95% CIs for PTSD in the original NKIR group, compared with those in the GP, were the highest at 4.91 (3.59-6.71). The HRs and 95% CIs in NKIR, compared with those in the GP, were 3.10 (2.90-3.30) for major depressive disorer, 2.27 (2.11-2.44) for anxiety and panic disorder, 2.03 (1.58-2.59) for bipolar disorder, 1.85 (1.53–2.24) for alcohol use disorder, and 1.89 (1.46-2.45) for schizophrenia. However, this association was significant for major depressive disorer, anxiety and panic disorder, alcohol use disorder, and PTSD in the PSM cohort.

**Table 4.** Subanalysis for Incidence of Individual Mental Disorders Before and After Propensity Score Matching^a^

Disorder	Original				Propensity score matching			
	Incidence of mental disorder, No. of cases		HR (95% CI)	*P* value	Incidence of mental disorder, No. of cases		HR (95% CI)	*P* value
	GP	NKIR			GP	NKIR		
Major depressive disorder	3127	2007	3.10 (2.90-3.30)	<.001	449	709	2.20 (1.95-2.47)	<.001
Anxiety and panic disorder	2976	1308	2.27 (2.11-2.44)	<.001	378	505	1.86 (1.62-2.13)	<.001
Attention- deficit/hyperactivity disorder	508	226	1.09 (0.90-1.32)	.38	90	71	0.94 (0.68-1.28)	.67
Alcohol use disorder	407	209	1.85 (1.53-2.24)	<.001	65	81	1.68 (1.21-2.36)	.002
Bipolar affective disorder	260	120	2.03 (1.58-2.59)	<.001	45	42	1.34 (0.87-2.05)	.18
Schizophrenia	225	110	1.89 (1.46-2.45)	<.001	41	29	0.99 (0.61-1.60)	.96
Posttraumatic stress disorder	95	118	4.91 (3.59-6.71)	<.001	15	29	2.53 (1.35-4.74)	.004

Abbreviations: GP, general population; HR, hazard ratio; NKIR, North Korean immigrants and refugees.

^a^ The multivariable Cox regression models were adjusted for age (continuous), sex, insurance premium level (continuous), region, Charlson Comorbidity Index score (continuous), and presence of disability. The GP group served as the reference for calculation of HRs.

## Discussion

The key finding of this cohort study was that the mental illness risk among NKIRs settled in South Korea was more than twice that of the GP, as shown by the nationwide database of the NHIS. Few studies have examined mental health risks among NKIRs. NKIRs often face a higher prevalence of mental illness compared with the GP of South Koreans. According to published studies on this population,^9^ the prevalence of anxiety was reported to be 43% for adults and 54% for adolescents,^10^ the prevalence of depression was 33% to 51%,^11,12^ and the prevalence of PTSD was reported to be as high as 52%.^13^ However, these studies have limitations in that they did not use control groups, and their findings were thus too difficult to generalize because they used convenience samples with tens or hundreds of people and self-report tools.

Migration is an established risk factor for the development of mental disorders.^14,15^ Our findings were consistent with this finding. The mental illness risk among NKIR individuals was more than twice that of GP individuals (HR = 2.12), and this was still significant after PSM (HR = 1.63) (Table 3).

Several global studies on immigrants and refugees have reported a high prevalence of depression, anxiety, psychotic disorders, and PTSD.^6^ Although there were some differences in the degree of study design, setting, or outcome measures, our study showed consistent results. Our study has some unique implications regarding the effect of migration on mental illness risk in a group sharing a common ethnic heritage (groups sharing a less widely spoken language or having few cultural differences).

There is still some controversy regarding high-risk and low-risk levels for substance use disorders (including those related to alcohol) among immigrant and refugee groups. A well-designed nationwide cohort study^16^ of a population of 1.2 million published in Sweden found that the risk of substance use disorder was low in immigrant and refugee groups. However, previous studies^17,18^ have indicated a high substance use disorder risk among immigrant and refugee populations. Our findings showed that alcohol use disorder risk was significantly high even after PSM in NKIR populations. As substance use issues are greatly influenced by individual countries’ policies and systems, we must strive to integrate the results of numerous variables (eg, ethnicity, region of origin, status of refugees, and current PTSD).

### Strengths and Limitations

Our study has several strengths. First, our results add some new information regarding the risk of mental illness occurrence in immigrant groups with low cultural heterogeneity, including language, compared with that in general population groups using claims data including all NKIRs residing in South Korea. Our findings have unique implications for determining mental illness risks for immigrants and refugees who already share the linguistic background of their new country. The lack of language barriers faced by these immigrants and refugees can minimize the controversy regarding the validity of such diagnoses in clinical practice. Second, regarding data, this study applied a strict operational definition with a sufficient 3-year washout period before assessing the mental illness occurrence rate. Third, this study included all age groups and targeted all psychiatric diagnoses. Often, PTSD, depression, and anxiety disorders have formed the main subjects of immigrant and refugee studies; studies on other mental disorders such as substance use disorder and ADHD have been extremely rare. However, our study included all psychiatric diagnoses with *ICD-10* F codes (Table 1).

Our study has several limitations. As the NHIS constituted the data source of our study, we encountered a lack of information on family members, occupation status, and any residence in a third country during migration. Similarly, as the claims data are based on *ICD-10* codes for administrative purposes, this study could not use *Diagnostic and Statistical Manual of Mental Disorders* diagnoses. Suicide is also an important area of interest for this population, but our study could not include it because of the lack of relevant valid information. Future research studies could investigate this factor by linking the cause of death with NHIS data.

## Conclusions

Assessing mental health risk in refugees and immigrants is an area in high demand, but data are still insufficient. In this cohort study, we observed the increased risk of stress-related psychiatric illness such as major depressive disorer, anxiety and panic disorder, alcohol use disorder, and PTSD among NKIR compared with South Korean citizens even after PSM.

## Notes

Supplement. **eFigure.** The Survival Probability for Each Group **eTable 1.** Study Population Observation Period Before and After PS Matching **eTable 2.** Incidence of Individual Mental Disorders and Hazard Ratios Before and After PS Matching

## References

[1] Dempster H, Hargrave K. Understanding public attitudes towards refugees and migrants. 2017. Accessed September 12, 2022. https://cdn.odi.org/media/documents/11600.pdf

[2] UNHCR. Global trends: forced displacement in 2019. 2022. Accessed August 25, 2022. https://www.unhcr.org/globaltrends2019/

[3] Bongaarts J. United Nations Department of Economic and Social Affairs, Population Division World family planning 2020: Highlights, United Nations publications, 2020. 46 p. Popul Dev Rev. 2020;46(4):857-858. doi:10.1111/padr.12377

[4] Blackmore R, Boyle JA, Fazel M, . The prevalence of mental illness in refugees and asylum seekers: a systematic review and meta-analysis. PLoS Med. 2020;17(9):e1003337. doi:10.1371/journal.pmed.1003337 32956381 PMC7505461

[5] Blackmore R, Gray KM, Boyle JA, . Systematic review and meta-analysis: the prevalence of mental illness in child and adolescent refugees and asylum seekers. J Am Acad Child Adolesc Psychiatry. 2020;59(6):705-714. doi:10.1016/j.jaac.2019.11.011 31778780

[6] Steel Z, Chey T, Silove D, Marnane C, Bryant RA, van Ommeren M. Association of torture and other potentially traumatic events with mental health outcomes among populations exposed to mass conflict and displacement: a systematic review and meta-analysis. JAMA. 2009;302(5):537-549. doi:10.1001/jama.2009.1132 19654388

[7] Fazel M, Wheeler J, Danesh J. Prevalence of serious mental disorder in 7000 refugees resettled in western countries: a systematic review. Lancet. 2005;365(9467):1309-1314. doi:10.1016/S0140-6736(05)61027-6 15823380

[8] Ministry of Unification. Settlement support for North Korean defectors in 2019. Accessed September 15, 2022. https://www.unikorea.go.kr/eng_unikorea/whatwedo/support/

[9] Lee Y, Lee M, Park S. Mental health status of North Korean refugees in South Korea and risk and protective factors: a 10-year review of the literature. Eur J Psychotraumatol. 2017;8(suppl 2):1369833. doi:10.1080/20008198.2017.1369833 29038687 PMC5632770

[10] Choi SK, Min SJ, Cho MS, Joung H, Park SM. Anxiety and depression among North Korean young defectors in South Korea and their association with health-related quality of life. Yonsei Med J. 2011;52(3):502-509. doi:10.3349/ymj.2011.52.3.502 21488195 PMC3101050

[11] Jeon B-H, Kim M-D, Hong S-C, . Prevalence and correlates of depressive symptoms among North Korean defectors living in South Korea for more than one year. Psychiatry Investig. 2009;6(3):122-130. doi:10.4306/pi.2009.6.3.122 20046386 PMC2796059

[12] Kim S-J, Kim H-H, Kim J-E, Cho S-J, Lee Y-J. Relationship between physical illness and depression in North Korean defectors. Korean J Psychosom Med. 2011;19(1):20-27.

[13] Lee YJG, Jun JY, Lee YJ, . Insomnia in North Korean refugees: association with depression and post-traumatic stress symptoms. Psychiatry Investig. 2016;13(1):67-73. doi:10.4306/pi.2016.13.1.67 26766948 PMC4701687

[14] Parrett NS, Mason OJ. Refugees and psychosis: a review of the literature. Psychosis. 2010;2(2):111-121. doi:10.1080/17522430903219196

[15] Hollander AC, Dal H, Lewis G, Magnusson C, Kirkbride JB, Dalman C. Refugee migration and risk of schizophrenia and other non-affective psychoses: cohort study of 1.3 million people in Sweden. BMJ. 2016;352:i1030. doi:10.1136/bmj.i1030 26979256 PMC4793153

[16] Harris S, Dykxhoorn J, Hollander A-C, Dalman C, Kirkbride JB. Substance use disorders in refugee and migrant groups in Sweden: a nationwide cohort study of 1.2 million people. PLoS Med. 2019;16(11):e1002944. doi:10.1371/journal.pmed.1002944 31689291 PMC6830745

[17] Hjern A, Allebeck P. Alcohol-related disorders in first- and second-generation immigrants in Sweden: a national cohort study. Addiction. 2004;99(2):229-236. doi:10.1046/j.1360-0443.2003.00562.x 14756715

[18] Bogic M, Ajdukovic D, Bremner S, . Factors associated with mental disorders in long-settled war refugees: refugees from the former Yugoslavia in Germany, Italy and the UK. Br J Psychiatry. 2012;200(3):216-223. doi:10.1192/bjp.bp.110.084764 22282430

