# Supplementary material for: Assessing Mental Illness Risk Among North Korean Refugees and Immigrants Resettled in South Korea
Source: JAMA Netw Open. 2022 Oct 19;5(10):e2236751. doi: 10.1001/jamanetworkopen.2022.36751 (PMC9582901; doi:10.1001/jamanetworkopen.2022.36751)
Supplement: Supplement. — eFigure. The Survival Probability for Each Group eTable 1. Study Population Observation Period Before and After PS Matching eTable 2. Incidence of Individual Mental Disorders and Hazard Ratios Before and After PS Matching [file jamanetwopen-e2236751-s001.pdf]

Supplemental Online Content

Lee R, Lee SM, Hong M, Oh IH. Assessing mental illness risk among North Korean refugees and immigrants resettled in South Korea. *JAMA Netw Open*. 2022;5(10):e2236751. doi:10.1001/jamanetworkopen.2022.36751

eFigure. The Survival Probability for Each Group

eTable 1. Study Population Observation Period Before and After PS Matching

eTable 2. Incidence of Individual Mental Disorders and Hazard Ratios Before and After PS Matching

This supplemental material has been provided by the authors to give readers additional information about their work.

© 2022 Lee R et al. *JAMA Network Open.*

eFigure. The Survival Probability for Each Group

GP, general population; NKMR, North Korean migrants and refugees

eFigure1 :The survival probability for each group

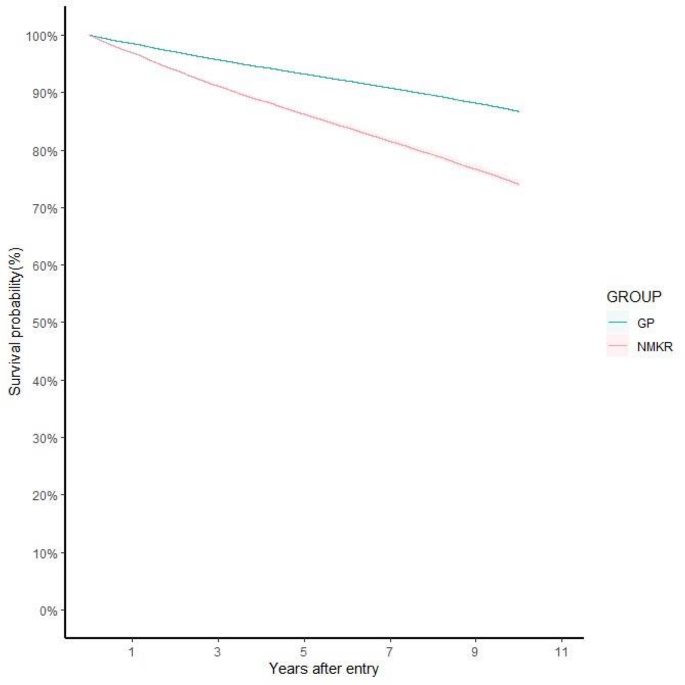

© 2022 Lee R et al. *JAMA Network Open.*

eTable 1. Study Population Observation Period Before and After PS Matching

	Original			PS matching		
	Average Period	Min	Max	Average Period	Min	Max
GP	3,651	1	4,147	3,651	2	4,104
NKMR	2,190	1	4,111	2,555	2	4,108

PS, propensity score; GP, general population; NKMR, North Korean migrants and refugees.

eTable 2. Incidence of Individual Mental Disorders and Hazard Ratios Before and After PS Matching

Disease	Original					PS matching				
	Incidence of mental disorder		HR	95% CI	P value	Incidence of mental disorder		HR	95% CI	P value
	GP (ref)	NKMR				GP (ref)	NKMR			
Schizophrenia	225	110	1.89	1.46-2.45	<0.001	41	29	0.99	0.61-1.60	0.96
Alcohol use disorder	407	209	1.85	1.53-2.24	<0.001	65	81	1.68	1.21-2.36	0.002
Drug use disorder	62	51	3.48	2.26-5.36	<0.001	7	25	4.73	2.04-11.00	<0.001
MDD	3,127	2,007	3.10	2.90-3.30	<0.001	449	709	2.20	1.95-2.47	<0.001
Dysthymia	204	100	2.32	1.77-3.05	<0.001	31	40	1.80	1.12-2.89	0.02

© 2022 Lee R et al. *JAMA Network Open.*

Bipolar affective disorder	260	120	2.03	1.58-2.59	<0.001	45	42	1.34	0.87-2.05	0.18
Anxiety panic disorder	2,976	1,308	2.27	2.11-2.44	<0.001	378	505	1.86	1.62-2.13	<0.001
Obsessive compulsive disorder	114	12	0.66	0.35-1.24	0.20	15	6	0.52	0.20-1.35	0.18
PTSD	95	118	4.91	3.59-6.71	<0.001	15	29	2.53	1.35-4.74	0.004
Dissociative disorder	23	8	1.29	0.52-3.17	0.58	5	1	0.27	0.03-2.36	0.24
Eating disorder	97	22	1.16	0.70-1.92	0.32	15	10	0.89	0.40-1.99	0.78
Autistic disorder	84	30	1.35	0.80-2.30	0.26	7	9	1.80	0.64-5.05	0.26
ADHD	508	226	1.09	0.90-1.32	0.38	90	71	0.94	0.68-1.28	0.67
Conduct disorder	60	34	1.30	0.78-2.15	0.31	14	9	0.80	0.34-1.85	0.60
Idiopathic intellectual disability	194	46	0.58	0.40-0.82	0.002	54	18	0.55	0.32-0.96	0.03
Borderline personality disorder	31	5	0.44	0.16-1.23	0.12	3	1	0.45	0.05-4.97	0.53
Other mental disorder	5,720	2,450	2.03	1.92-2.14	<0.001	730	967	1.76	1.59-1.94	<0.001

PS, propensity score; GP, general population; NKMR, North Korean migrants and refugees; MDD, major depressive disorder; PTSD, posttraumatic stress disorder; ADHD, attention deficit/hyperactivity disorder.

© 2022 Lee R et al. *JAMA Network Open.*
